# TRIM50 Suppresses Pancreatic Cancer Progression and Reverses the Epithelial-Mesenchymal Transition *via* Facilitating the Ubiquitous Degradation of Snail1

**DOI:** 10.3389/fonc.2021.695740

**Published:** 2021-09-09

**Authors:** Rongkun Li, Lili Zhu, Yangxizi Peng, Xiaoxin Zhang, Chunhua Dai, Dejun Liu

**Affiliations:** ^1^Institute of Oncology, Affiliated Hospital of Jiangsu University, Zhenjiang, China; ^2^State Key Laboratory of Oncogenes and Related Genes, Shanghai Cancer Institute, Ren Ji Hospital, School of Medicine, Shanghai Jiao Tong University, Shanghai, China; ^3^Department of Rheumatology and Immunology, Changhai Hospital, Naval Medical University, Shanghai, China; ^4^Jiangsu Key Laboratory of Medical Science and Laboratory Medicine, School of Medicine, Jiangsu University, Zhenjiang, China; ^5^Department of Biliary-Pancreatic Surgery, Ren Ji Hospital, School of Medicine, Shanghai Jiao Tong University, Shanghai, China

**Keywords:** TRIM50, tumor growth and metastasis, EMT, Snail1, pancreatic cancer

## Abstract

Emerging evidence suggests that the tripartite motif (TRIM) family play important roles in tumor development and progression. Tripartite motif-containing 50 (TRIM50) is a member of the TRIM family, but little is known regarding its expression and potential functional roles in cancer. In this study, we first analyzed the expression pattern and clinical significance of TRIM50 in pancreatic cancer and found that TRIM50 expression is significantly reduced in pancreatic cancer tissues and its downregulation is associated with poor survival for pancreatic cancer patients. Functionally, TRIM50 overexpression in pancreatic cancer cells decreases their proliferation and motility capabilities and reverses the epithelial-mesenchymal transition (EMT) process, whereas TRIM50 depletion had the opposite effects. Mechanically, TRIM50 directly interacts with Snail1, a key regulator of EMT, and acts as an E3 ubiquitin ligase to target Snail1 for ubiquitous degradation. The function of TRIM50 in suppressing cell migration and EMT depends on TRIM50-promoted Snail1 degradation. In conclusion, our findings identify TRIM50 as a tumor suppressor that inhibits pancreatic cancer progression and reverses EMT *via* degrading Snail1 and provide new insights into the progression of pancreatic cancer.

## Introduction

Pancreatic cancer is a lethal malignancy with a dismal 5-year survival rate of no more than 8% (1). Despite significant improvements in the diagnosis and therapy of pancreatic cancer, overall survival remains relatively poor due to its late diagnosis, early metastasis, and resistance to radiotherapy and chemotherapy (2, 3). Unfortunately, the underlying mechanism for pancreatic cancer growth and metastasis remain poorly understood. Therefore, an improved understanding of the molecular mechanisms involved in the development and progression of pancreatic cancer is an urgent need for designing effective interventional strategies and prolonging patient life.

Tripartite motif (TRIM) family proteins have more than 80 members and are characterized by a series of conserved domains, including a RING domain, one or two B-boxes, and a coiled-coil domain at the N terminus. The presence of a RING domain indicates that TRIM proteins can function as E3 ubiquitin ligases. Numerous studies have shown that TRIM proteins play important roles in many aspects of carcinogenesis, such as cell cycle, apoptosis, proliferation, differentiation, metabolism, and immune response (4). Tripartite motif-containing protein 50 (TRIM50), as a member of the TRIM family, was first identified as an E3 ubiquitin ligase in Williams-Beuren syndrome and reported to promote the formation of sophisticated canaliculi and microvilli during acid secretion in parietal cells (5, 6). In addition, another study demonstrated that TRIM50 interacts with HDAC6 and regulates P62 degradation (7). It is well documented that many TRIM family proteins are positively or negatively involved in the initiation and progression of various cancers as E3 ubiquitin ligases. However, TRIM50 has been seldom reported about its biological function in cancer. Up to now, TRIM50 has only been shown to act as a tumor suppressor in hepatocellular carcinoma and ovarian cancer (8, 9).

The epithelial-to-mesenchymal transition (EMT) is a distinctive morphogenic process that plays central roles in development, wound healing, and stem cell behavior and is often activated in cancer progression and fibrosis (10). Cancer cells undergoing EMT exhibit both morphological changes and molecular alterations, as characterized by the decreased expression of epithelial markers and the increased expression of mesenchymal markers. During EMT, cancer cells acquire more aggressive features, including cell plasticity, invasiveness, and resistance to apoptosis (11). Several transcription factors including Snail family transcriptional repressor 1 (Snail1) are known as pivotal regulators of EMT (12). It has been reported that the abundance of Snail1 is finely regulated by ubiquitination established by several E3 ubiquitin ligase complexes and rapid proteasomal degradation in cells (13, 14).

In our present study, we analyzed the expression and clinical significance of TRIM50 in pancreatic cancer and explored the roles of TRIM50 in the development and progression of pancreatic cancer. We found that TRIM50 expression was significantly downregulated in pancreatic cancer tissues and negatively associated with malignant characteristics and clinical survival of pancreatic cancer patients. TRIM50 overexpression inhibited pancreatic cancer cell proliferation, migration, and invasion *in vitro* and suppressed pancreatic cancer growth and distant metastasis *in vivo*, while TRIM50 knockdown had the opposite effects *in vitro*. TRIM50 interacted with Snail1 and promoted its ubiquitous degradation, thus reversing EMT. Furthermore, TRIM50 suppressed the malignant phenotypes of pancreatic cancer dependent on its inhibition of Snail1.

## Materials and Methods

### Data Mining Using the Gene Expression Omnibus and the Gene Expression Profiling Interactive Analysis 2

The expression of *TRIM50* at mRNA level was analyzed using the microarray gene expression data sets obtained from the GEO database (http://www.ncbi.nlm.nih.gov/geo/) with the accession codes GSE15471, GSE16515, GSE28735, GSE32676, GSE60980, GSE62165, GSE62452, GSE71729, and GSE102238 and GEPIA2 database (http://gepia.cancer-pku.cn/index.html) with an overview of the RNA sequencing data based on the Cancer Genome Atlas (TCGA) and the Genotype-Tissue Expression (GTEx) projects.

### Cell Culture

Human pancreatic cancer cell lines, including AsPC-1, BxPC-3, Capan-1, CFPAC-1, MIA PaCa-2, PANC-1, Patu8988, and SW1990, were preserved in Shanghai Cancer Institute, Ren Ji Hospital, School of Medicine, Shanghai Jiao Tong University. AsPC-1, BxPC-3, CFPAC-1, and Patu8988, were maintained in RPMI 1640 medium (Hyclone) and Capan-1, MIA PaCa-2, PANC-1, and SW1990 were maintained in Dulbecco’s modified Eagle’s medium (DMEM, Hyclone), supplemented with 10% (v/v) fetal bovine serum (FBS, Hyclone), 100 Units/ml of penicillin, and 100 μg/ml of streptomycin at 37°C in a 5% CO_2_ incubator.

### Immunohistochemistry Staining and Scoring

A paraffin-embedded tissue microarray (OD-CT-DgPan01-006), containing 90 pairs of pancreatic cancer tissues and adjacent non-tumor tissues, was purchased from Shanghai Outdo Biotech Inc (Shanghai, China). Paraffin sections were baked for 30 minutes at 60°C for deparaffinization and rehydration. After inhibition of endogenous peroxidase activity for 30 minutes with methanol containing 0.3% H_2_O_2_, the sections were blocked with 10% BSA at room temperature (RT) for 1 hour, incubated with anti-TRIM50 (1:50 dilution, Abcam, ab174880) overnight at 4°C, labeled with HRP second antibody at RT for 1 hour, developed in DAB solution and countered with hematoxylin. The scoring of TRIM50 expression was carried out based on both the ratio and intensity of the staining as previously described (15). For IHC analysis on xenograft tumors for Ki-67 staining (1:200 dilution, Abcam, ab16667), tumor specimens were fixed in 4% paraformaldehyde, embedded in paraffin, and subjected to IHC staining.

### RNA Extraction and Quantitative Real-Time Polymerase Chain Reaction

Total RNA was isolated from cultured cell lines using Trizol reagent (Takara, Japan). Complementary DNA (cDNA) synthesis was conducted with a PrimeScript RT-PCR kit (Takara, Japan). qRT-PCR was performed using 2 × SYBR Green qPCR Master Mix (Bimake.cn) according to the manufacturer’s instructions with specific primers on the ABI7500 instrument. The sequences of primers were as follows: *18sRNA* (forward: 5’-TGCGAGTACTCAACACCAACA-3’, reverse: 5’-GCATATCTTCGGCCCACA-3’), *TRIM50* (forward: 5’-CTGGTGGATGAGGAGAAGGC-3’, reverse: 5’-TCCGGATGAACTTGTGGTGG-3’), *CDH1* (forward: 5’-TCAGGGTCGGTTGGAAATC-3’, reverse: 5’- AATGCCGCCATCGCTTAC-3’), *CDH2* (forward: 5’-GTCAGCAGAAGTTGAAGAAATAGTG-3’, reverse: 5’-GCAAGTTGATTGGAGGGATG-3’), *Vimentin* (forward: 5’- TCTTCCAAACTTTTCCTCCC, reverse: 5’-AGTTTCGTTGATAACCTGTCC-3’), *Snail1* (forward: 5’-TGCGTCTGCGGAACCTG-3’, reverse: 5’-GGACTCTTGGTGCTTGTGGA-3’), *Snial2* (forward: 5’-TGTGACAAGGAATATGTGAGCC-3’, reverse: 5’-TGAGCCCTCAGATTTGACCTG-3’), *Twist1* (forward: 5’-CTGAGCAAGATTCAGACCCTCA-3’, reverse: 5’-TCCATCCTCCAGACCGAGAAG-3’), *Twist2* (forward: 5’-ACAGCAAGAAGTCGAGCGAA-3’, reverse: 5’-GCAGCGTGGGGATGATCTTG-3’), *ZEB1* (forward: 5’-ACTGTTTGTAGCGACTGGATT-3’, reverse: 5’-TAAAGTGGCGGTAGATGGTA-3’), and *ZEB2* (forward: 5’-TTCCATTGCTGTGGGCCTT-3’, reverse:5’-TTGTGGGAGGGTTACTGTTGG-3’). *18sRNA* was used as an internal control.

### Western Blotting Analysis

Western blotting analysis was conducted as described previously in our study (16). Total cellular proteins were harvested in IP lysis buffer (Beyotime, Shanghai, China) with a cocktail of proteinase and phosphatase inhibitors (Bimake.cn) and were measured by a BCA protein assay kit (Thermo Fisher Scientific, USA). Protein lysates were separated by sodium dodecyl sulfate-polyacrylamide gel electrophoresis (SDS-PAGE) and transferred to nitrocellulose (NC) membrane (Millipore, USA). Then the membranes were blocked with 5% skimmed milk in Tris-Buffered saline (TBS) for 1 hour at RT and incubated with primary antibodies overnight at 4°C followed by incubation with DyLight fluorescent dye labelled secondary antibodies for 1 hour at RT. Finally, immunoblot signals were detected using an Odyssey Imaging System (LI-COR Biosciences, USA). Primary antibodies used in this analysis were listed as follows: TRIM50 (1:100 dilution, Abcam, ab174880), E-cadherin (1:5000, Proteintech, 20874-1-AP), N-cadherin (1:2000, Proteintech, 22018-1-AP), Vimentin (1:2000, Proteintech, 10366-1-AP), Snail1 (1:1000, Proteintech, 13099-1-AP), Snail2 (1:2000, Proteintech, 12129-1-AP), Twist1 (1:1000, Proteintech, 25465-1-AP), Twist2 (1:1000, Proteintech, 11752-1-AP), ZEB1 (1:1000, Proteintech, 21544-1-AP), ZEB2 (1:2000, Proteintech, 14026-1-AP), and β-actin (1:5000, MultiSciences, ab008).

### Plasmid Constructs and Transfection

For TRIM50 overexpression, the TRIM50-overexpressing and vector control (termed as vector) plasmids were purchased from GeneCopoeia (EX-OL09564-lx304, Guangzhou, China). Pancreatic cancer cells, BxPC-3 and Patu8988, were transfected with the plasmids for 24 hours following the manufacturer’ instructions and then were selected with 10 μg/ml blasticidin for 2 weeks. For co-immunoprecipitation (CoIP) analysis and rescue experiment, the plasmids expressing HA-tagged TRIM50 (NM_001281451.1) and Snail1 (NM_005985.4) were constructed using the pcDNA3.1(+) vector as described in our previous study (15). The plasmids were transfected into the indicated pancreatic cancer cells using the jetPRIME transfection reagent (Polyplus transfection Inc, Illkirch, France) according to the manufacturer’s instructions. Briefly, the cells at 50-60% confluency in 10 mm culture dishes were exposed to 3 μg of plasmid using 1:2 ratio of DNA (μg): jetPRIME (μl) reagent. The culture medium was replaced after 6 hours and the efficiency of TRIM50 or Snail1 overexpression was confirmed after 24 hours by qRT-PCR and western blotting.

### siRNA Transfection

TRIM50 siRNAs or negative control (termed as siTRIM50-NC) were used to transfect PANC-1 and SW1990 cells as directed by the manufacturer (GenePharma Inc., Shanghai, China). Briefly, the cells were transfected with siRNAs at a concentration of 50 nM using Lipofectamine 2000 (Invitrogen, Carlsbad, CA, USA) in optiMEM medium (Gibco) for 6 hours and then incubated in complete medium for another 48 hours. The sequences of TRIM50 siRNA were as follows: siTRIM50-1 (sense: 5’-GGCCCUUAGAAGGCGCAUUTT-3’, antisense: 5’-AAUGCGCCUUCUAAGGGCCTT-3’); siTRIM50-2 (sense 5’-CCGGGUGUACGAAGCCUUUTT-3’, antisense: 5’-AAAGGCUUCGUACACCCGGTT-3’). The efficiency of siRNA transfection was measured at 48-hour post-transfection by qRT-PCR and western blotting.

### Rescue of TRIM50 Depletion

Six silent mutations were introduced into the sequence targeted by the siTRIM50-1 in order to make it resistant to this siRNA. The mutated TRIM50 cDNA was synthetized by shanghaiGenerayBiotechCo.,Ltd, and then the mutated TRIM50 plasmid was constructed using the pcDNA3.1(+) vector. For rescue experiments, PANC-1 and SW1990 cells were transfected with TRIM50 or control siRNAs as described above. 16 hours later, the culture medium was replaced, and 1 µg mTRIM50-expressing plasmid or empty vector was transfected into the cells for 24 hours using the jetPRIME transfection reagent as described above. The presence of endogenous and mutated TRIM50 protein was examined by western blotting.

### Cell Proliferation Assay

Cell proliferation was measured using the cell counting kit-8 (CCK-8) assay following the standard procedure. In brief, cells with a density of 1-2 × 10^3^ cells per well were seeded in 96-well plates. At the indicated time points, the CCK-8 reagent (10 μl/well) was added and the reaction system was incubated at 37°C for an additional 1 hour. The relative proliferation of cells was obtained at 450 nm absorbance.

### Colony Formation Assay

1 × 10^3^ cells in single-cell suspension were seeded per 6-well plate. The culture medium was replaced every 3 days. 2 weeks later, the formed colonies were washed twice with PBS, fixed with 4% paraformaldehyde for 30 minutes, and stained with 0.2% crystal violet for 1 hour. The colonies with more than 50 cells were counted under a microscope.

### Cell Apoptosis Assay

Cell apoptosis was detected using FITC-Annexin V Apoptosis Detection Kit (BD Bioscience, USA). Cells were seeded in 6-well plates and incubated overnight, and then the medium was replaced by FBS-free medium. 24 hours later, the cells were harvested with trypsin and washed with phosphate-buffered saline (PBS) twice. Then, the cells were stained with FITC-labled Annexin V and propidium iodide (PI) for 15 minutes at RT. Finally, cell apoptosis was detected by flow cytometric analysis.

### Cell Migration and Invasion Assays

Migration and invasion assays were carried out with 8.0-μm pore inserts (Millipore, Bedford, MA, USA) in 24-well plates. The lower chamber was filled with 750 μl medium containing 10% FBS. For migration assay, 5 × 10^4^ cells in medium without serum were seeded into the upper chamber and the cells were allowed to migrate for 24 hours. For invasion assay, 1 × 10^5^ cells in medium without serum were seeded into the upper chamber coated with Matrigel (BD Biosciences, Franklin Lakes, NJ, USA) and the cells were allowed to invade for 48 hours. After removal of the non-migrated and -invaded cells, the remaining cells were fixed with 4% paraformaldehyde and stained with 0.2% crystal violet. Migrated and invaded cells were photographed in 5 randomly selected fields and counted.

### Wound Healing Assay

Cells were seed in 6-well plates and were allowed to grow till almost 100% confluency. Then, wounds were scratched using a P200 peptide tip and fresh medium free of FBS was added. Images were captured at 0 hour and 48 hour using inverted microscope (Ziess, Germany). Cell motility was analyzed by measuring the distance of wound closure using ImageJ software.

### Animal Experiments

For tumor growth model, 2 × 10^6^ TRIM50-overexpressing BxPC-3 and vector cells were resuspended in 100 μl PBS and subcutaneously injected into the back of the BALB/c mice (male, 4- to 6-week old, 18-20g, 5 mice per group). 5 weeks later, the mice were killed and the tumors were resected, weighted, embedded in paraffin, and subjected to IHC staining of TRIM50 and Ki-67.

For lung metastasis model, 1 × 10^6^ TRIM50-overexpressing BxPC-3 and vector cells were resuspended in 100 μl PBS were injected into the tail vein of the BALB/c mice (male, 4- to 6-week old, 18-20g, 5 mice per group). 5 weeks later, the mice were killed and their lung tissues were resected, embedded in paraffin, and subjected to hematoxylin and eosin (H&E) staining.

For liver metastasis model, 2 × 10^6^ TRIM50-overexpressing BxPC-3 and vector cells were resuspended in 25 μl PBS were injected into the spleen of the BALB/c mice (male, 4- to 6-week old, 18-20g, 5 mice per group). 5 weeks later, the mice were killed and their liver tissues were resected, embedded in paraffin, and subjected to hematoxylin and eosin (H&E) staining.

The mice used in this study were manipulated and housed according to the protocols approved by the East China Normal University Animal Care Commission. All the mice received humane care according to the criteria outlined in the Guide for the Care and Use of Laboratory Animals prepared by the National Academy of Sciences and published by the National Institutes of Health.

### Snail1 Protein Stability With Cycloheximide

Snail1 protein stability was evaluated by treating BxPC-3 and Patu8988 cells stably transfected with TRIM50 or PANC-1 cells transiently transfected with specific TRIM50 siRNAs with CHX (20 μg/ml) for 1 and 3 hours as indicated. Then the cell lysis was harvested followed by western blotting and the scale of the bands was analyzed by ImageJ software.

### CoIP

Cell lysates were harvested as described in western blotting and incubated with anti-HA (2 µg, Millipore, 05-904), and anti-IgG (as a negative control, 2 µg, Abcam, ab200699) with rotation for 30 minutes at RT, followed by incubation with Pierce Protein-A/G Magnetic Beads (Thermo Fisher Scientific, USA, #88803) for 30 minutes at RT. Finally, immuno-complexes were washed three times with TBS-T and then resuspended in 1 × SDS-PAGE sample buffer for western blotting analysis.

### Ubiquitination Assay

BxPC-3 and Patu8988 cells stably transfected with TRIM50 or PANC-1 cells transiently transfected with specific TRIM50 siRNAs were transfected with V5-ubiquitin plasmids using the jetPRIME transfection reagent as described above. The cells were then incubated with 10 μM MG132 for 6 hours. Then total proteins were extracted, followed by CoIP performed using anti-Snail1. The protein complexes were subsequently subjected to western blotting using anti-ubiquitination antibody (1:1000, Proteintech, 10201-2-AP) to evaluate the ubiquitination level of Snail1.

### Statistical Analysis

Statistical analyses were carried out using SPSS 19.0 for windows (IBM Corporation, USA) and GraphPad Prism 7 software (San Diego, CA). The association between TRIM50 expression and clinicopathological characteristics was analyzed by the Chi-square test and Fisher’s exact probability method. Overall survival was evaluated by Kaplan-Meier method and the log-rank test. Univariate and multivariate analyses based on the Cox proportional hazards regression model were performed to identify independent prognostic factors when conditions are applicable. Quantitative variables were analyzed by the two-tailed Student t-test or analysis of variance. Difference with *P* < 0.05 between groups was considered statistically significant.

## Results

### TRIM50 Expression Is Frequently Downregulated in Multiple Pancreatic Cancer Cohorts

**Figure 1:**
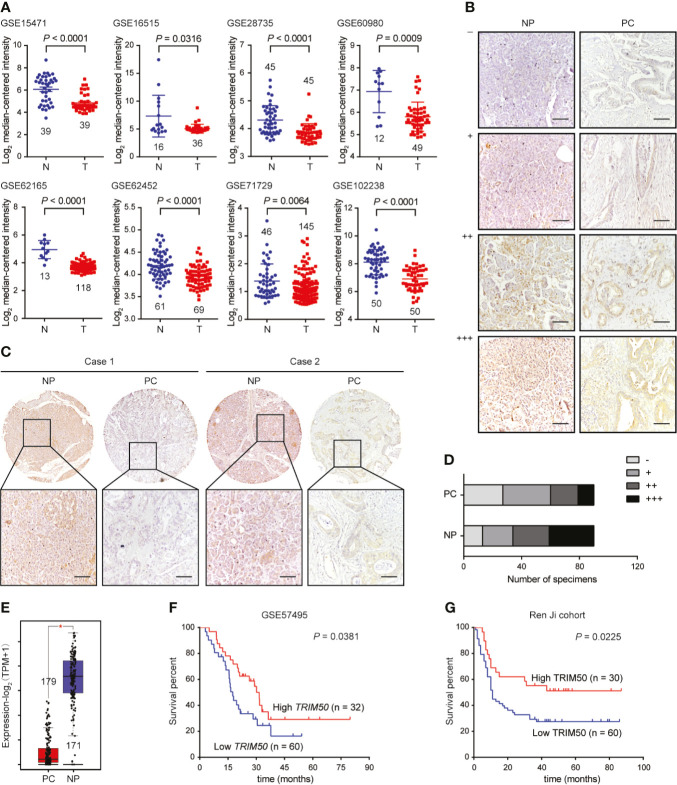
TRIM50 expression in pancreatic cancer tissues and cell lines and its association with prognosis. **(A)** Comparison of *TRIM50* mRNA expression level in pancreatic cancer (T) and normal pancreas (N) tissues based on the data derived from multiple independent GEO datasets. **(B)** IHC staining of TRIM50 in pancreatic cancer (PC) and normal pancreas (NP) tissues. Representative images of NP and PC tissues with different TRIM50 immunoreactivity displaying no (-), weak (+), moderate (++), or strong (+++). Scale bar: 50 μm. **(C, D)** Comparison of TRIM50 expression in paired NP and PC tissues. Scale bar: 50 μm. **(E)** Comparison of *TRIM50* mRNA expression level in PC and NP tissues based on the data derived from TCGA cohort and GTEx. **(F, G)** Kaplan-Meier curves showing significant association of TRIM50 expression with pancreatic cancer patients’ survival in GSE57495 **(F)** and Ren Ji cohorts **(G)**.

To investigate the expression pattern of TRIM50 in pancreatic cancer, we first analyzed the expression of *TRIM50* at mRNA level in pancreatic cancer and normal tissues based on the data from GEO database. As shown in **Figure 1A**, *TRIM50* expression was significantly decreased in pancreatic cancer tissues compared with normal pancreas tissues as revealed by multiple GEO datasets. To validate the GEO observations, the expression of TRIM50 at protein level in 90 pairs of pancreatic cancer and adjacent nontumor tissues was assessed by IHC staining (**Figure 1B**). As a result, similar results were obtained from IHC staining experiments, showing TRIM50 downregulation in tumor tissues (**Figure 1C**). Our results also revealed low expression of TRIM50 (- or +) in the majority of pancreatic tumors examined, whereas high expression of TRIM50 (++ or +++) in the majority of nontumor specimens (**Figure 1D**). The downregulated expression of *TRIM50* was further confirmed by the transcriptomic profiles in a combination of TCGA and GTEx **(** **Figure 1E**).

Then, the association between TRIM50 expression and clinicopathological characteristics was analyzed in our cohort of pancreatic cancer tissues. TRIM50 expression was found to be negatively correlated with tumor size, lymphatic metastasis, and TNM stage shown in **Table 1**. However, there was no correlation between TRIM50 expression and age, gender, tumor grade, vascular invasion, as well as distant metastasis. Furthermore, we evaluated the prognostic significance of TRIM50 expression in GSE57495 dataset and our cohort, both of which showed that pancreatic cancer patients with high TRIM50 expression had more favorable prognosis than those with low TRIM50 expression (**Figures 1F, G** **)**. Univariate and multivariate Cox regression analyses were also conducted to confirm the prognostic value of TRIM50. Unexpectedly, TRIM50 expression was not an independent prognostic predictor for the survival of pancreatic patients (**Supplementary Figure 1**). Taken together, these data suggest that TRIM50 may act as a tumor suppressor in pancreatic cancer.

**Table 1:** Correlations between TRIM50 expression and clinicopathologic parameters in pancreatic cancer patients.

Clinicopathological parameters	Total	Expression of TRIM50		*p* value
		Low	High	
**Age (years)**				
< 60	41	26	15	0.655
≥ 60	49	34	15	
**Gender**				
Male	57	41	16	0.174
Female	33	19	14	
**Tumor size** ^#^				
≤ 5 cm	55	31	24	0.005*
> 5 cm	34	29	5	
**Tumor grade**				
I	1	1	0	0.501
II	56	36	20	
III	22	17	5	
IV	1	1	0	
**Lymphatic metastasis** ^#^				
Absent	48	26	22	0.004*
Present	33	28	5	
**Vascular invasion**				
Absent	52	32	20	0.227
Present	38	28	10	
**Distant metastasis**				
Absent	88	58	30	0.551
Present	2	2	0	
**TNM stage** ^#^				
I	40	20	20	0.015*
II	47	38	9	
IV	2	2	0	

*Significant difference in statistics.

^#^Tumor size and TNM stage assessments of 89 patients, lymphatic metastasis assessment of 81 patients, since the data about these parameters were not retrieved in all specimens.

### TRIM50 Overexpression Inhibits Cell Proliferation and Induces Cell Apoptosis in Pancreatic Cancer

**Figure 2:**
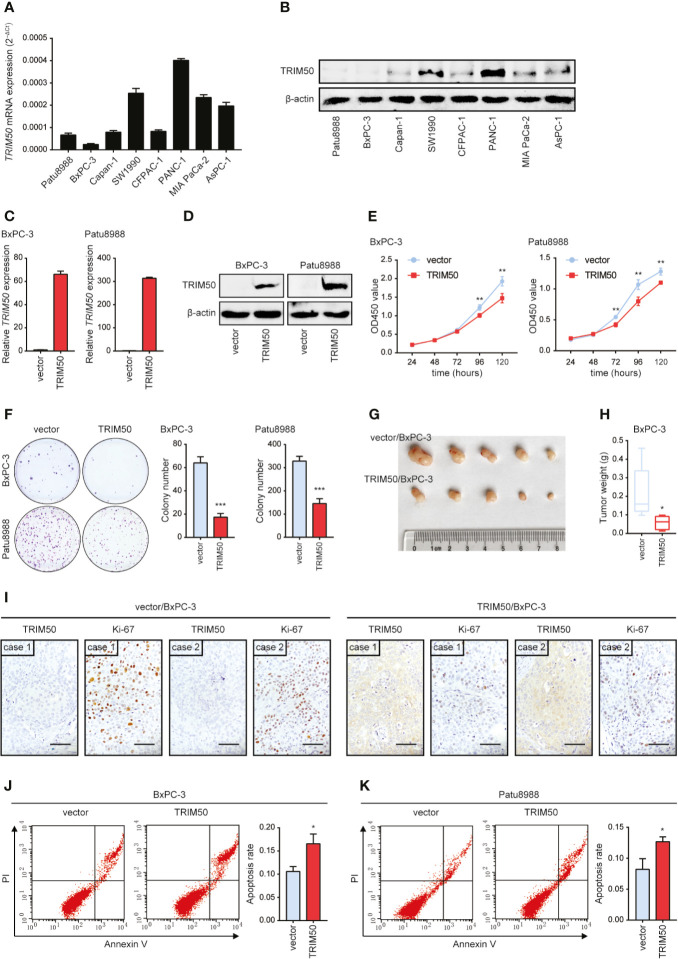
TRIM50 overexpression suppresses tumor growth and induces cell apoptosis in pancreatic cancer. **(A, B)** Pancreatic cancer cell lines were assayed for relative TRIM50 expression by qRT-PCR **(A)** and western blotting **(B). (C, D)** The efficiency of TRIM50 overexpression confirmed by qRT-PCR **(C)** and western blotting **(D)**. **(E, F)** The effect of TRIM50 overexpression on cell proliferation revealed by CCK-8 **(E)** and colony formation **(F)** assays. **(G)** Nude mice (5 per group) were subcutaneously injected with TRIM50-overexpressing and vector control cells; gross xenografts were shown 35 days after injection. **(H)** Weights of primary subcutaneous tumors were determined in **(G)**. **(I)** IHC staining of TRIM50 and Ki-67 in TRIM50-overexpressing and vector control subcutaneous xenografts. Scale bar: 50 μm. **(J, K)** The effect of TRIM50 overexpression on cell apoptosis of BxPC-3 **(J)** and Patu8988 **(K)** cells revealed by Annexin V/PI-labeled flow cytometric assay. *P < 0.05, **P < 0.01, and ***P < 0.001.

To identify whether TRIM50 is involved in pancreatic cancer suppression, we firstly measured the expression of TRIM50 in human pancreatic cancer cell lines (**Figures 2A, B** **)**. Next, we chose two cell lines with relatively lower TRIM50 expression, BxPC-3 and Patu8988, for overexpression. The overexpression efficiency was confirmed by qRT-PCR (**Figure 2C**) and western blotting (**Figure 2D**). We showed that TRIM50 overexpression significantly inhibited cell proliferation compared with vector control in BxPC-3 and Patu8988 cells (**Figure 2E**), which was confirmed by the results of the colony formation assay (**Figure 2F**). Then, we used the nude mouse xenograft model to validate the effect of TRIM50 overexpression on tumor growth *in vivo*. As expected, the weight of tumors in TRIM50 overexpression group (TRIM50/BxPC-3) was markedly decreased compared with the control group (vector/BxPC-3) (**Figures 2G, H** **)**. In addition, IHC staining of Ki-67 was performed to evaluate tumor growth and showed that the TRIM50-overexpressing group had lower proliferative indexes than the vector control group (**Figure 2I**). Additionally, TRIM50 overexpression induced pancreatic cancer cell apoptosis shown by flow cytometry analysis, which may be a reason for decreased tumor growth (**Figures 2J, K** **)**. Collectively, the above data demonstrate that TRIM50 inhibits cell proliferation and tumor growth and induces cell apoptosis in pancreatic cancer.

### TRIM50 Overexpression Suppresses Cell Migration, Cell Invasion, and EMT in Pancreatic Cancer

**Figure 3:**
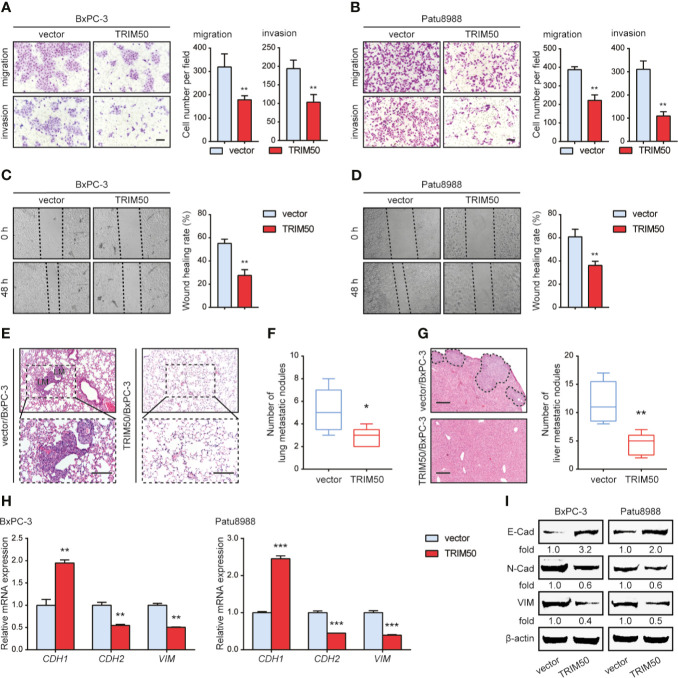
TRIM50 overexpression inhibited cell migration, cell invasion, and lung metastasis in pancreatic cancer. **(A, B)** The effect of TRIM50 overexpression on cell migration and invasion of BxPC-3 **(A)** and Patu8988 **(B)** cells revealed by Transwell assays. Scale bar: 50 μm. **(C, D)** The effect of TRIM50 overexpression on cell mobility of BxPC-3 **(C)** and Patu8988 **(D)** cells revealed by wound healing assay. **(E)** TRIM50-overexpressing and vector control BxPC-3 cells were injected into the tail vein of nude mice (5 per group). Representative H&E images of lung metastasis (LM). Scale bar: 50 μm. **(F)** Quantification of lung metastasis after 5 weeks after injection. **(G)** TRIM50-overexpressing and vector control BxPC-3 cells were injected into the spleen of nude mice (5 per group). Representative H&E images of liver metastasis (left panel), quantification of liver metastasis after 5 weeks after injection (right panel). Scale bar: 200 μm. **(H, I)** The effect of TRIM50 overexpression on the expression of EMT-related markers, E-Cadherin (E-Cad), N-Cadherin (N-Cad), and Vimentin (VIM), confirmed by qRT-PCR **(H)** and western blotting **(I)**. *P < 0.05, **P < 0.01, and ***P < 0.001.

We further evaluated the effects of TRIM50 on cell migration and invasion by Transwell and wound healing assays. The results showed that TRIM50 overexpression significantly reduced the migratory and invasive capacity of BxPC-3 (**Figure 3A**) and Patu8988 (**Figure 3B**) cells. Similar results were obtained from the wound healing assay, TRIM50 overexpression significantly decreased the wound healing rate of BxPC-3 (**Figure 3C**) and Patu8988 (**Figure 3D**) cells. To corroborate the function of TRIM50 in pancreatic cancer metastasis further, we injected TRIM50-overexpressing BxPC-3 and vector control cells into the tail vein or spleen of nude mice. At day 35 after injection, we found that the mice harboring TRIM50-overexpressing BxPC-3 cells had less metastatic nodules in their lungs (**Figures 3E, F** **)** or livers (**Figure 3G**) than control mice. Altogether, these results show that TRIM50 suppresses cell migration and distant metastasis of pancreatic cancer.

As described above, TRIM50 overexpression inhibited pancreatic cancer cell migration and invasion, we explored the mechanisms associated with this change in phenotype. Given that EMT is closely associated with tumor metastasis (17) and that many members of the TRIM family, including TRIM50, participate in cancer progression *via* regulating EMT (8, 9, 18), we determined whether TRIM50 affects the EMT process of pancreatic cancer cells. Indeed, TRIM50 overexpression increased the expression of E-cadherin, an epithelial marker of EMT, and decreased the expression of N-cadherin and Vimentin, mesenchymal markers of EMT, at both mRNA (**Figure 3H**) and protein (**Figure 3I**) levels, indicating that TRIM50 reverses the EMT process.

### TRIM50 Depletion Promotes Cell Proliferation, Cell Migration, Cell Invasion, and EMT in Pancreatic Cancer

**Figure 4:**
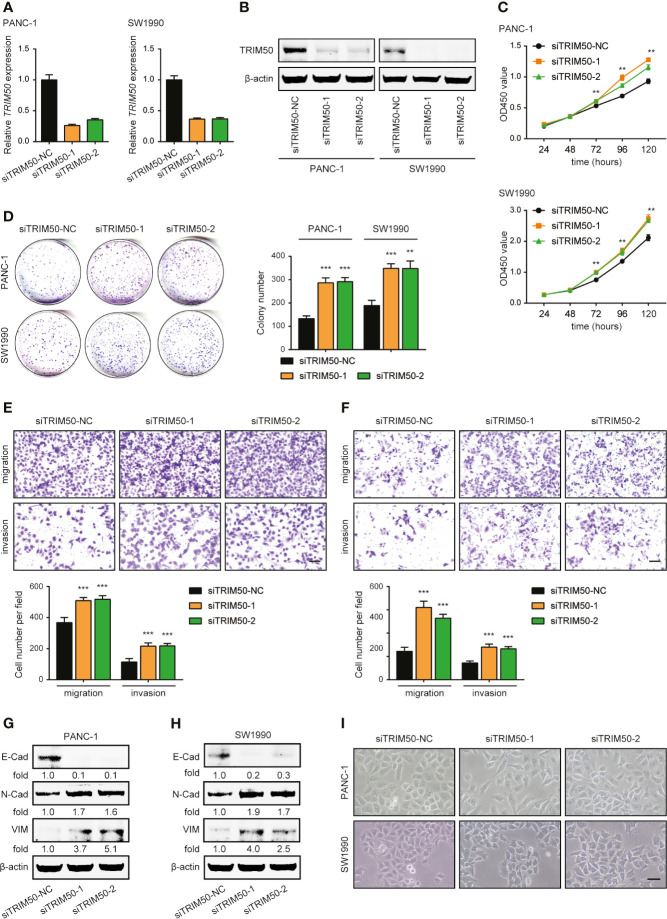
Depletion of TRIM50 promotes cell proliferation, migration, and invasion and induces EMT in pancreatic cancer. **(A, B)** The efficiency of TRIM50 depletion confirmed by qRT-PCR **(A)** and western blotting **(B)**. **(C, D)** The effect of TRIM50 depletion on cell proliferation revealed by CCK-8 **(C)** and colony formation **(D)** assays. **(E, F)** The effect of TRIM50 depletion on cell migration and invasion of PANC-1 **(E)** and SW1990 **(F)** cells revealed by Transwell assays. Scale bar: 50 μm. **(G, H)** Western blotting confirming the decreased expression of E-Cadherin (E-Cad) and increased expression of N-Cadherin (N-Cad) and Vimentin (VIM) following TRIM50 depletion in PANC-1 **(G)** and SW1990 **(H)** cells. **(I)** PANC-1 and SW1990 cells transfected with TRIM50 or control siRNAs were photographed. Scale bar: 50 μm. **P < 0.01 and ***P < 0.001.

Two cell lines with relatively higher TRIM50 expression, PANC-1 and SW1990, were selected for depletion, the efficiency of which was also confirmed by qRT-PCR and western blotting (**Figures 4A, B** **)**. Conversely, TRIM50 depletion exhibited significant promoting effects on cell viability revealed by CCK-8 (**Figure 4C**) and colony formation (**Figure 4D**) assays, as well as on cell migration and invasion revealed by Transwell assays (**Figures 4E, F** **)**. Moreover, TRIM50 depletion induced the EMT process, as demonstrated by the decreased expression of E-cadherin and the increased expression of N-cadherin and Vimentin (**Figures 4G, H** **)**. Indeed, TRIM50 depletion resulted in the transition to a dispersed morphology both in PANC-1 and SW1990 cells (**Figure 4I**). Collectively, the results further confirm TRIM50 as an antioncogene in pancreatic cancer.

To ascertain the specific roles of TRIM50 in EMT-associated phenotypes, we ectopically re-expressed TRIM50 in TRIM50 depletion cells (**Supplementary Figure 2A**). The results showed that rescue of TRIM50 significantly restored the decreased cell proliferation (**Supplementary Figure 2B**) and invasion (**Supplementary Figure 2C**), and reversed EMT (**Supplementary Figure 2D**) caused by TRIM50 depletion in PANC-1 and SW1990 cells, further confirming the requirement of TRIM50 in EMT-associated phenotypes of pancreatic cancer.

### TRIM50 Reverses EMT *via* Destabilizing Snail1 in Pancreatic Cancer Cells

**Figure 5:**
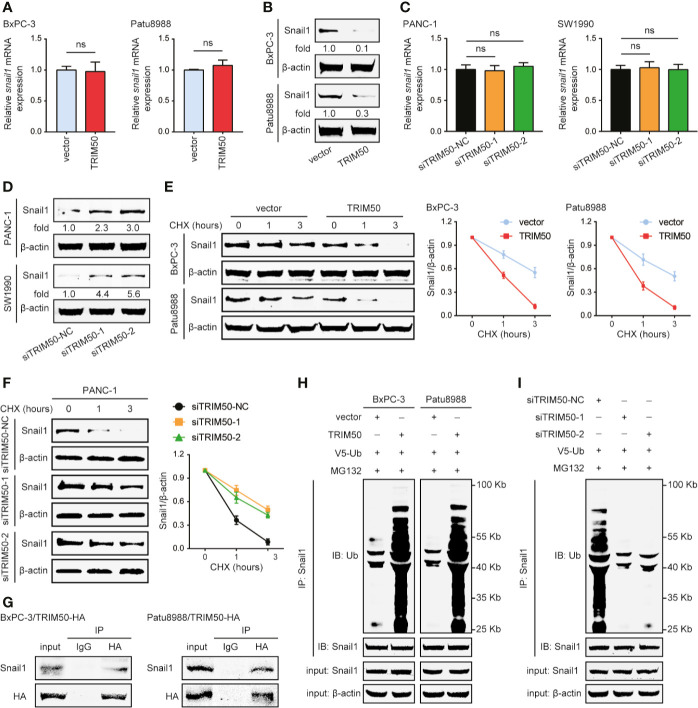
TRIM50 interacts with Snail1 for its ubiquitous degradation. **(A, B)** The effect of TRIM50 overexpression on Snail1 expression level at mRNA **(A)** and protein **(B)** levels. **(C, D)** The effect of TRIM50 depletion on Snail1 expression level at mRNA **(C)** and protein **(D)** levels. **(E)** TRIM50 overexpression destabilized Snail1 protein expression. Western blotting showing Snail1 in TRIM50-overexpressing and vector control cells as indicated (left panel). Cells were harvested at the indicated times following cycloheximide (CHX) treatment to inhibit new protein synthesis. Quantitation of Snail1 normalized to the loading control and expressed to 0 hour (right panel). **(F)** TRIM50 depletion stabilized Snail1 protein expression. Western blotting showing Snail1 in TRIM50 depletion and vector control cells as indicated (left panel). Quantitation of Snail1 conducted as TRIM50 overexpression (right panel). **(G)** BxPC-3 and Patu8988 cells were transfected with HA-tagged TRIM50 plasmids and subjected to CoIP of TRIM50-HA with endogenous Snail1 48 hours later. TRIM50 was immunoprecipitated with an HA antibody, Snail1 bound to TRIM50 was determined using a Snail1 antibody. **(H)** BxPC-3 and Patu8988 cells with or without TRIM50 overexpression were transfected with V5-ubiquitin (V5-Ub) plasmids, pretreated with 10 μM MG132 for 6 hours and subjected to anti-Snail1 IP followed by anti-ubiquitin (Ub) with western blotting analysis 48 hours later. **(I)** PANC-1 cells with or without TRIM50 depletion mediated by siRNAs were subjected to experiments as described for **(H)**. ns, no significance.

Next, we investigated the underlying mechanism by which TRIM50 regulates EMT. Due to the key roles of EMT-related transcription factors in the EMT process, we examined whether TRIM50 regulates EMT *via* affecting the expression of EMT-related transcription factors, including Snail1, Snail2, Twist1, Twist2, ZEB1, and ZEB2. TRIM50 overexpression decreased, whereas TRIM50 depletion increased Snail1 expression at protein level (**Figures 5B, D** **)**, but neither TRIM50 overexpression nor depletion had significant implications on *Snail1* expression at mRNA level (**Figures 5A, C** **)**. However, the expression of Snail2, Twist1, Twist2, ZEB1, and ZEB2 were not significantly affected by TRIM50 overexpression nor depletion at both mRNA and protein levels (**Supplementary Figures 3A–D**). These data indicate that TRIM50 may reverse EMT *via* specially regulating the expression of Snail1 through post-transcriptional mechanisms.

It has been reported that many TRIM proteins, as E3 ubiquitin ligases, are involved in cancer initiation and progression *via* interaction with substrate proteins to regulate their stability. So, we first tested the effects of TRIM50 on Snail1 protein stability following inhibition of new protein synthesis by CHX treatment. Snail1 half-time was shortened in TRIM50-overexpressing cells (**Figure 5E**), but prolonged in TRIM50 depletion cells (**Figure 5F**). The proteasome inhibitor MG132 blocked TRIM50-mediated regulation on Snail1 (**Figures 5H, I** **)**, suggesting that Snail1 downregulation induced by TRIM50 depends on the ubiquitin-proteasome way. Next, we detected whether TRIM50 interacts with Snail1, and confirmed an interaction between TRIM50 and Snail1 by co-immunoprecipitation performed with anti-HA antibody (**Figure 5G**). Based on the findings above, we examined whether TRIM50 could promote the ubiquitination of Snail1 as an E3 ubiquitin ligase. As expected, TRIM50 overexpression considerably increased the level of ubiquitination of Snail1 in BxPC-3 and Patu8988 cells (**Figure 5H**). Conversely, TRIM50 depletion remarkably decreased the level of ubiquitination of Snail1 in PANC-1 cells (**Figure 5I**). In summary, these data show that TRIM50 interacts with Snail1 for its ubiquitous degradation, resulting in a decreased Snail1 stability.

**Figure 6:**
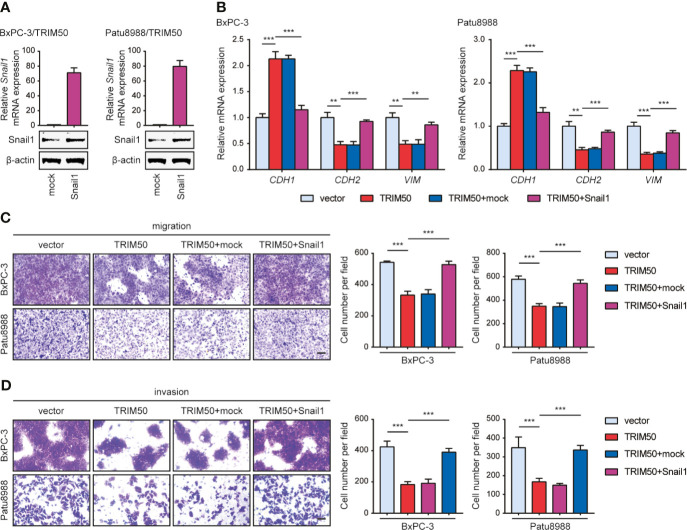
Snail1 is responsible for the effects of TRIM50 on cell migration, invasion, and EMT. **(A)** TRIM50-overexpressing BxPC-3 and Patu8988 cells were transfected with Snail1 and vector pcDNA3.1(+) (termed as mock) plasmids. The efficiency of Snail1 expression was confirmed by qRT-PCR (upper panel) and western blotting (lower panel). **(B)** Re-expression of Snail1 largely or completely abrogated the inhibiting effects of TRIM50 on EMT by examining the expression of EMT-related markers at mRNA level. **(C, D)** Re-expression of Snail1 largely or completely abrogated the inhibiting effects of TRIM50 on cell migration **(C)** and cell invasion **(D)** revealed by Transwell assays. Scale bar: 50 μm. **P < 0.01 and ***P < 0.001.

Furthermore, we tested whether TRIM50 inhibits EMT, cell migration, and cell invasion dependent on Snail1. To this end, we expressed Snail1 in TRIM50-overexpressing cells as shown in **Figure 6A**. Snail1 expression successfully reversed the inhibiting effects of TRIM50 on EMT by examining the expression of EMT-related markers (**Figure 6B**). In addition, Snail1 expression restored the migratory and invasive capability of TRIM50-overexpressing to that of vector control cells (**Figures 6C, D** **)**. These data confirm that TRIM50 suppresses the malignant phenotypes of pancreatic cancer in a Snail1-dependent manner.

## Discussion

Increasing evidence has shown that TRIM proteins are involved in the development and progression of multiple human cancer types, as either oncogenes or tumor-suppressors. It has been reported that some members of the TRIM family are dysregulated and implicated in the pathogenesis of pancreatic cancer (19–23). However, the expression pattern and role of TRIM50 in pancreatic cancer remains unknown. In our present study, we found that the expression of TRIM50 was significantly downregulated in pancreatic cancer tissues, which was closely associated with tumor size, lymphatic metastasis, and TNM stage. Similar decreased TRIM50 expression was also observed in hepatocarcinoma (8). Furthermore, pancreatic cancer patients with low TRIM50 expression had a worse prognosis than those with high TRIM50 expression. The findings suggest that TRIM50 may exert antioncogenic roles in pancreatic cancer. A recent study shows that p53 can bind to the promoter of TRIM67 to induce significant upregulation of TRIM67 and the methylation level of TRIM67 promoter is significantly increased, finally resulting in TRIM67 downregulation in colorectal cancer (24). However, critical regulators of TRIM50 expression in pancreatic cancer are not involved in our study, which needs to be studied further.

In our study, a series of *in vitro* and *in vivo* experiments showed that TRIM50 overexpression suppressed cell proliferation and tumor growth, and induced cell apoptosis. In addition, depletion of TRIM50 further confirmed its anticarcinogenic effect in pancreatic cancer. Several studies have demonstrated that many TRIM family proteins regulate tumor growth and cell apoptosis *via* controlling the abundance and activity of p53 (24–26). For instance, TRIM59 promotes gastric carcinogenesis *via* interacting with p53 and promoting its ubiquitination and degradation (26); TRIM67 interacts with p53, resulting in inhibition of MDM2-mediated p53 degradation, finally activates p53 to suppress the initiation and progression in colorectal cancer (24). In contrast with it, neither TRIM50 overexpression nor depletion had effects on the p53 level in pancreatic cancer cells (data not shown). The molecular mechanism by which TRIM50 inhibits pancreatic cancer growth warrants further study.

Furthermore, we showed that TRIM50 inhibited pancreatic cancer invasiveness and distant metastasis and reversed the EMT process, which was supported by the roles of TRIM67 as tumor suppressor in breast cancer (27) and lung cancer (28). In breast cancer, TRIM67 blocks TGF-β-mediated EMT by binding to and promoting the ubiquitination of SMAD3 (27). In lung cancer, haploinsufficiency of Trim62 synergizes with a K-RasG12D mutation to promote invasiveness and disrupt three-dimensional morphogenesis, which is closely associated with EMT (28).

Snail1 is well known as a key regulator of EMT. Overexpression of Snail1 has been found in pancreatic cancer, which correlates with nodal metastasis and distant metastasis and predicts poor clinical outcome for pancreatic cancer (29, 30). Snail1 contributes to the metastasis, chemoresistance, and maintenance of stem cell-like phenotype in pancreatic cancer (31, 32). Snial1 is a labile protein and is under the control of constant ubiquitination and degradation mediated by E3 ubiquitin ligases, such as FOXO11, FBW7, and SBSP2 (33–36). We found that E3 ubiquitin ligase TRIM50 interacted with Snail1, targeted it to ubiquitination and degradation, and finally destabilized it in pancreatic cancer. In addition, TRIM50 reversed EMT and suppressed cell migration and invasion depending on its inhibition of Snail1. A similar result was obtained in hepatocellular carcinoma, which showed that TRIM50 directly binds with Snail1 for its K-48 linked ubiquitous degradation, thus reversing Snail1-mediated EMT process (8). However, the domains that mediate the interaction between TRIM50 and Snail1, as well as the ubiquitination sites and subcellular localization of Snail1 mediated by TRIM50 in pancreatic cancer warrant further investigations.

In this study, we noticed that TRIM50 localized mainly in the cytoplasm of normal pancreas and pancreatic cancer tissues. Given that the cytosolic localization of TRIM50, we speculated that TRIM50 mainly targets cytosolic Snail1 for degradation and subsequently decreases the nuclear translocation of Snail1, finally resulting in decreased transcription activity of Snail1, which still need a series of experiments to test. Simultaneously, nuclear TRIM50 was often observed in normal pancreas tissues, but rarely in pancreatic cancer tissues, suggesting possible different roles for TRIM50 under physiological and pathological conditions.

In summary, our study shows that decreased TRIM50 expression is positively correlated with malignant behaviors and predicts a poor prognosis in pancreatic cancer. TRIM50 suppresses cell proliferation and tumor growth and induces cell apoptosis. Moreover, TRIM50 inhibits cell invasiveness and induces EMT in a Snail1-dependent manner *via* interacting with it for its ubiquitination and degradation. Our findings that TRIM50 acts as an E3 ubiquitin ligase for Snail1 will provide new therapeutic strategies for human malignancies with abnormal accumulation of Snail1.

## Data Availability Statement

The original contributions presented in the study are included in the article/**Supplementary Material**. Further inquiries can be directed to the corresponding authors.

## Ethics Statement

The studies involving human participants were approved by the Ethics Committee of the Institutional Review Board of Ren Ji Hospital, School of Medicine, Shanghai Jiao Tong University. The patients/participants provided their written informed consent to participate in this study. The animal study was reviewed and approved by East China Normal University Animal Care Commission.

## Author Contributions

DL and CD designed the study and revised the final manuscript. RL, LZ, and YP carried out the *in vitro* and *in vivo* experiments, analyzed the results of the *in vitro* and *in vivo* experiments, and finished the manuscript writing. XZ performed IHC staining and analyzed the data. All authors contributed to the article and approved the submitted version.

## Funding

The study was supported by Science and Technology Projects of Affiliated Hospital of Jiangsu University (NO. jdfyRC2020003).

## Conflict of Interest

The authors declare that the research was conducted in the absence of any commercial or financial relationships that could be construed as a potential conflict of interest.

## Conflict of Interest

The authors declare that the research was conducted in the absence of any commercial or financial relationships that could be construed as a potential conflict of interest.

## Publisher’s Note

All claims expressed in this article are solely those of the authors and do not necessarily represent those of their affiliated organizations, or those of the publisher, the editors and the reviewers. Any product that may be evaluated in this article, or claim that may be made by its manufacturer, is not guaranteed or endorsed by the publisher.

## Supplementary Material

Supplementary Figure 1 Univariate analysis showing the association between TRIM50 expression and pancreatic cancer survival.

Supplementary Figure 2 Rescue of TRIM50 recovers its antioncogenic roles in pancreatic cancer. **(A)** Western blotting analysis of TRIM50 and mutated TRIM50 (mTRIM50) proteins was performed with extracts from PANC-1 and SW1990 cells transfected with control siRNA or siTRIM50-1 and either mTRIM50-expressing plasmid or empty vector. **(B–D)** Rescue of TRIM50 recovered the inhibiting effects of TRIM50 on cell proliferation **(B)**, cell invasion **(C)**, and the expression of EMT markers **(D)**. Scale bar: 50 μm. ***P* < 0.01 and ****P* < 0.001.

Supplementary Figure 3 The expression of EMT-related transcription factors followed by TRIM50 overexpression or depletion. **(A, B)** The effect of TRIM50 overexpression **(A)** or depletion **(B)** on the expression of EMT-related transcription factors at mRNA level. **(C, D)** The effect of TRIM50 overexpression **(C)** or depletion **(D)** on the expression of EMT-related transcription factors at protein level. **P* < 0.05; ns, no significance.
