# Supplementary figures and images for: TRIM50 Suppresses Pancreatic Cancer Progression and Reverses the Epithelial-Mesenchymal Transition via Facilitating the Ubiquitous Degradation of Snail1
Source: Front Oncol. 2021 Sep 9;11:695740. doi: 10.3389/fonc.2021.695740 (PMC8458909; doi:10.3389/fonc.2021.695740)

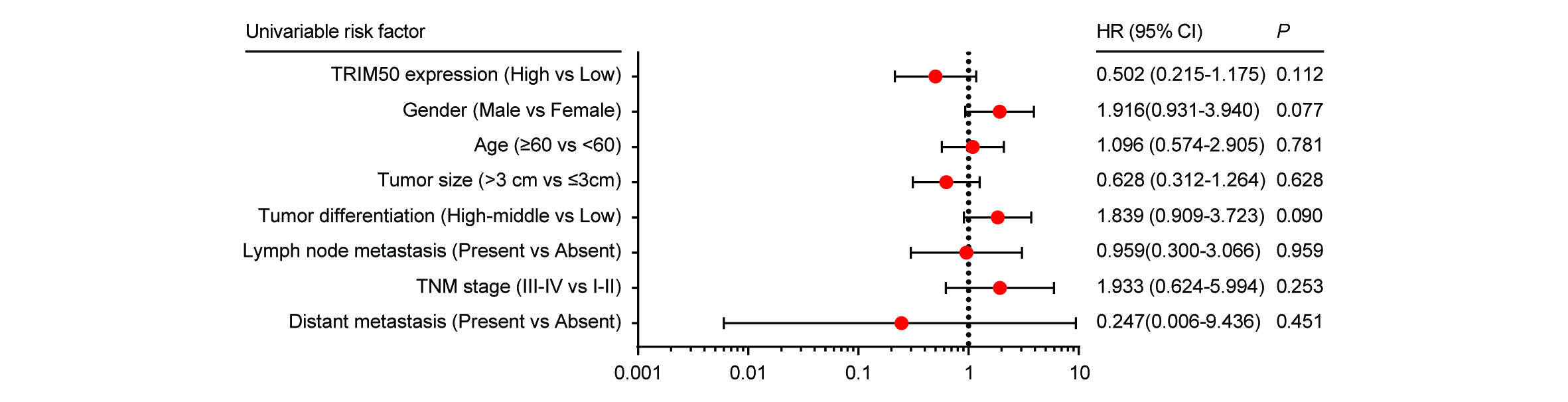

Supplement: Supplementary Figure 1 — Univariate analysis showing the association between TRIM50 expression and pancreatic cancer survival. [file Image_1.tif]

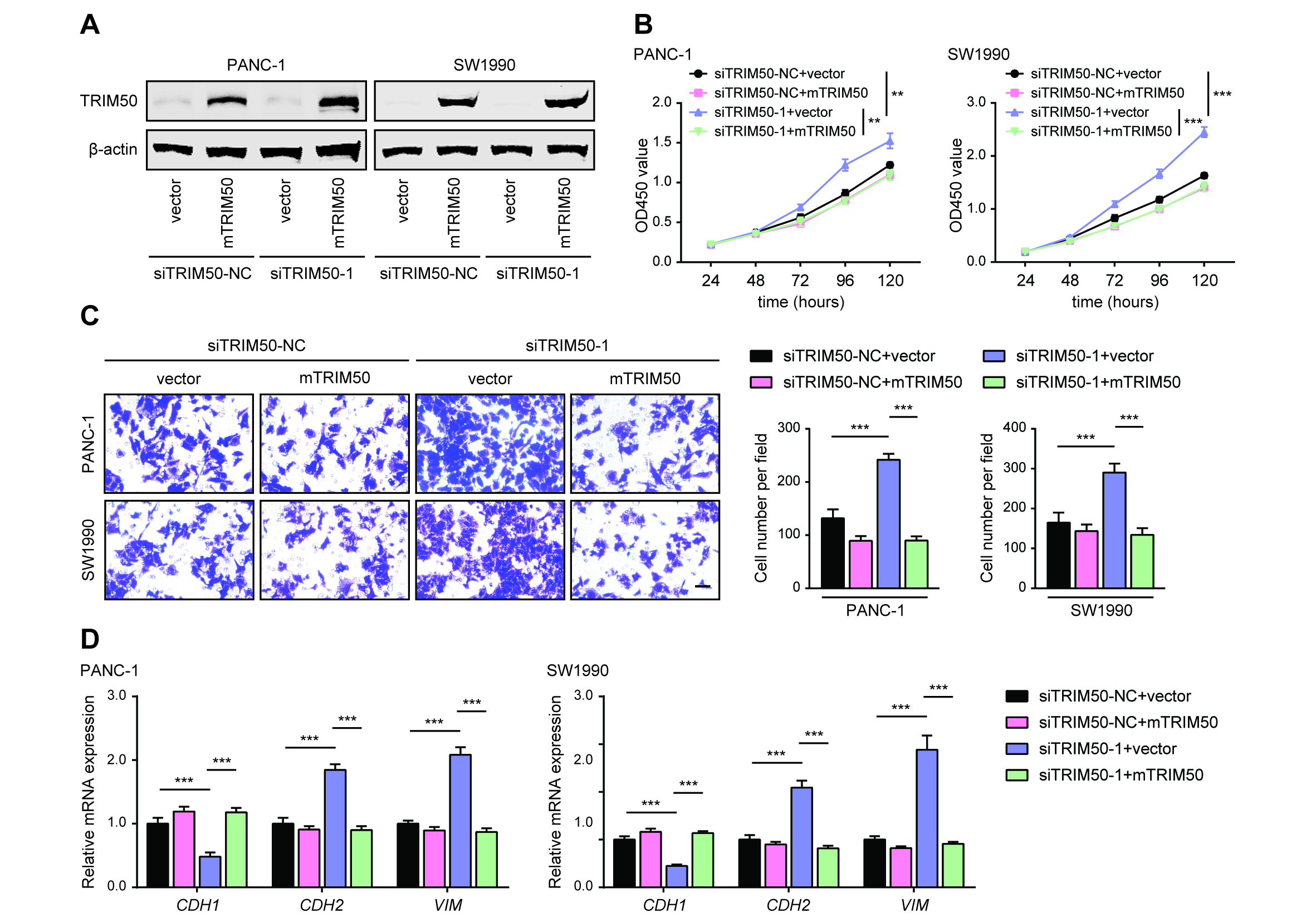

Supplement: Supplementary Figure 2 — Rescue of TRIM50 recovers its antioncogenic roles in pancreatic cancer. (A) Western blotting analysis of TRIM50 and mutated TRIM50 (mTRIM50) proteins was performed with extracts from PANC-1 and SW1990 cells transfected with control siRNA or siTRIM50-1 and either mTRIM50-expressing plasmid or empty vector. (B–D) Rescue of TRIM50 recovered the inhibiting effects of TRIM50 on cell proliferation (B), cell invasion (C), and the expression of EMT markers (D). Scale bar: 50 μm. **P < 0.01 and ***P < 0.001. [file Image_2.tif]

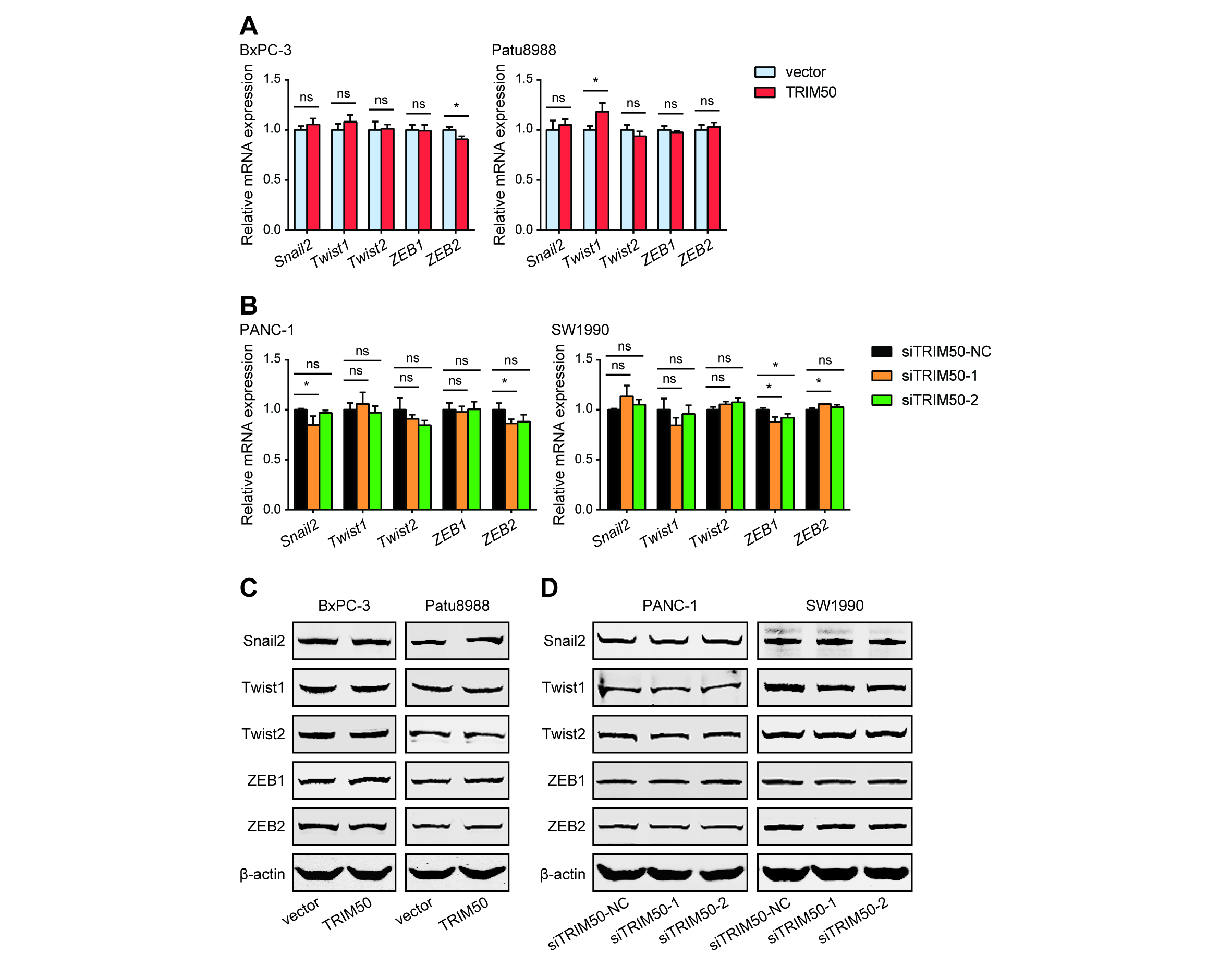

Supplement: Supplementary Figure 3 — The expression of EMT-related transcription factors followed by TRIM50 overexpression or depletion. (A, B) The effect of TRIM50 overexpression (A) or depletion (B) on the expression of EMT-related transcription factors at mRNA level. (C, D) The effect of TRIM50 overexpression (C) or depletion (D) on the expression of EMT-related transcription factors at protein level. *P < 0.05; ns, no significance. [file Image_3.tif]
